# Application of Activity Trackers among Nursing Home Residents—A Pilot and Feasibility Study on Physical Activity Behavior, Usage Behavior, Acceptance, Usability and Motivational Impact

**DOI:** 10.3390/ijerph17186683

**Published:** 2020-09-14

**Authors:** Tina Auerswald, Jochen Meyer, Kai von Holdt, Claudia Voelcker-Rehage

**Affiliations:** 1 Institute of Human Movement Science and Health, Faculty of Behavioural and Social Sciences, Chemnitz University of Technology, Thüringer Weg 11, 09126 Chemnitz, Germany; tina.auerswald@hsw.tu-chemnitz.de; 2 OFFIS—Institute for Information Technology Oldenburg, Escherweg 2, 26121 Oldenburg, Germany; meyer@offis.de (J.M.); kai.vonholdt@offis.de (K.v.H.); 3 Department of Neuromotor Behavior and Exercise, University of Münster, Horstmarer Landweg 62 b, 48149 Münster, Germany

**Keywords:** activity tracker, long-term care, physical activity, sedentary behavior, long-term use

## Abstract

The aim of this study was to assess physical activity and sedentary behavior, as well as the usage behavior, usability, acceptance, and motivational impact of an applied activity tracker among nursing home residents. Physical activity and usage behavior were measured among 22 residents (68 to 102 years) by use of a commercial activity tracker worn during waking hours for 77 days on average. Usability, acceptance, and motivational impact of the tracker were examined using an adapted questionnaire. Participants walked, on average, 1007 ± 806 steps per day and spent, on average, more than 9 h (77.2% of their waking time) sedentary. The average steps/day increased significantly within the first five weeks of wearing the activity tracker. The acceptance rate was high (94.4%). The tracker was used for 65.4% of the individual study period, and usage behavior did not significantly change during the first five wearing weeks. Participants with a usage time of ≥50% walked significantly more steps per day than those with a lower usage. Overall, we were able to reveal that the residents were highly inactive and sedentary. The results support the feasibility of a long-term application of activity trackers to assess or even increase physical activity behavior.

## 1. Introduction

In the European Union, the number of people aged 80 years and older is projected to grow from 4.9% in 2016 to 13% in 2070 [1]. Accordingly, forecasts predict an increase in public expenditure on long-term care from 1.6% to 2.7% of the gross domestic product between 2016 and 2070 [1]. In Germany, the number of care-dependent elderly, such as nursing home residents, is expected to increase by 47.4% up to 2030 [2].

Physical activity (PA), such as daily walks, has been shown to promote older adults’ health, quality of life, cognition, and physical performance [3,4,5,6,7,8], while sedentary behavior has been shown to increase the risk for chronic diseases, disability, physical frailty, and even early death [9,10,11,12]. Thus, PA is a promising approach to maintain health and independence into old age and to reduce health resource use and costs [13]. Despite these findings, older adults (especially nursing home residents) are considered the least active group [14,15,16]. Recently, a few studies examined PA patterns in ambulatory or long-term care settings, and if so, only for a short period of time. Research showed that persons in need of care spend more than 80% of their waking time lying down or sitting [17,18,19]. Buckinx and colleagues revealed that this population walks, on average, less than 1700 steps per day [20]. Even though there is some evidence for PA patterns of nursing home residents, research is still sparse and accurate quantification of nursing home residents’ PA is necessary [21]. This would help to derive consensus guidelines, as well as exercise intervention studies for this predominantly multi-morbid and frail population.

Overall, measuring PA in nursing home residents constitutes a challenge [22]. PA level is often measured subjectively by standardized PA questionnaires or diaries that are used to calculate the estimated energy expenditure of a person spent during PA (e.g., “International Physical Activity Questionnaire”, IPAQ) [23]. Due to cognitive, sensory, physical, and medical restrictions of nursing home residents, this approach is limited and another method for assessing PA should be considered [22,24]. Less often, PA is evaluated objectively by use of activity trackers or pedometers that assess, for example, number of steps or energy expenditure [17,20]. Objective measures typically have higher levels of accuracy and validity than subjective ones and, particularly for nursing home residents, have the advantage that they are less influenced by cognitive impairments. Objective monitoring instruments, like activity trackers, are becoming increasingly widespread and are used more often for assessment and interventions in younger and even older community-dwelling populations [25,26,27]. For also assessing PA in nursing home residents, activity trackers might be a feasible objective device as they are, for example, easy to use and inexpensive [28,29]. Despite these positive findings, such devices are rarely used in inpatient care facilities, like nursing homes, and so far, only a few studies have addressed the application of activity trackers in long-term care facilities by investigating (1) the relationship between physical frailty and daily PA [20], (2) the relationship between PA and sedentary behavior [18,19], (3) the accuracy of used activity trackers [21], and (4) the ease of use and toleration of the used activity trackers [30].

Furthermore, research has shown that activity trackers might be applied as an interventional approach, such that only its usage can improve the PA behavior and, in turn, the health status of older people (i.e., PA, body mass index (BMI), blood pressure) [7,25,31,32,33]. Even for nursing home residents, activity trackers might be used to support or improve their health status, besides just tracking PA behavior. However, the potential benefits of activity trackers for nursing home residents depend on whether the older adults actually use them. Therefore, empirical research about the usability and acceptance of activity trackers by older adults living in long-term care settings is needed. Currently, usage behavior, acceptance, and/or usability of activity trackers have been mainly investigated in younger or community-dwelling older adults [34,35,36,37,38,39,40,41,42]. In nursing home settings, research on acceptance and/or usability of activity trackers is much sparser, and current results are inconsistent [19,30]. So far, Cohen-Mansfield and colleagues have investigated the feasibility of using pedometers in nursing home settings, and found that the devices were easy to use and well-tolerated [30]. On the contrary, Reid and colleagues showed poor diary completion and loss of monitors when using an activity tracker (activePAL3^TM^) by residential aged care residents [19]. 

With regard to the state of research, there is currently a lack of studies investigating the activity levels of nursing home residents and their use and acceptance of activity trackers, especially over a longer period of time. Therefore, the first aim of this pilot and feasibility study was to examine the daily PA and sedentary behavior of nursing home residents with respect to wearing period (entire period, first versus fifth week, during summer). Moreover, we were interested in whether there were differences in PA and sedentary behavior with respect to gender and age, as well as usage behavior, cognitive status, and past and current activity behavior. Concerning the potential future application of activity trackers to increase the health behavior for this specific population, the second aim of this study was to determine the nursing home residents’ usage behavior, usability, acceptance, and motivational impact of such devices.

## 2. Materials and Methods 

### 2.1. Participants

Thirty elderly residents from four different nursing homes in the area of Chemnitz, Germany (22 women, 8 men) between 68 and 102 years of age participated in this pilot study. The data were collected in the period from January to September 2019. Recruitment and eligibility was based upon consultation of nursing staff, nursing documentation, and surveys in the context of the projects “Prevention in Stationary Care” and “Prevention and Occupational Health in Long-Term Care” (PROCARE), which focused on prevention in long-term care settings [43]. Written informed consent was obtained from each participant or the respective legal guardian. Inclusion criteria were: (1) being older than 60 years, (2) the ability to walk independently with or without a walking aid, and (3) the ability to understand and execute simple instructions. Excluded were participants who: (1) used a wheelchair, (2) were bedridden, (3) had severe cognitive impairments according to patient file or Montreal Cognitive Assessment (MoCA; severe dementia, cutoff = “<10”) [44], (4) precluded informed consent, or (5) were unwilling to participate. Eligibility criteria were assessed by senior management, nursing staff, and nursing documentation. The study was conducted within the framework of two projects which were approved by the respective ethics committee (Ethics committee of the Faculty of Behavioural and Social Sciences, Chemnitz University of Technology, Germany; Ethics Committee of the Hamburg Chamber of Physicians, Germany, registration number PV5762). All study participants were fully informed about the study and provided informed consent for study participation and further analysis of the assessed data following the declaration of Helsinki.

A total of five participants (16.7%) were excluded from PA data analysis because of a wearing time of the activity tracker of less than 10 days. Two participants (6.7%) lost the activity tracker during the study period. One participant (3.3%) deceased during the study period. Thus, a final total of 22 participants were included in data analysis (mean age 86.41 ± 9.32, range 68–102; 72.7% women).

### 2.2. Procedure and Measures

Age and BMI were collected from patient file data. Cognitive status was assessed by use of the Montreal Cognitive Assessment (MoCA; no dementia = “>23”, mild dementia = “18–23” or moderate dementia = “10–17”, *n* = 9) [44] or collected by staff survey (“no dementia”, “mild dementia”, “moderate dementia”, *n* = 11). Current participation in nursing home activities (never, rarely, frequently, or daily activity; 4-point Likert scale) and past activity level (never, rarely, occasionally, or regularly; 4-point Likert scale) were recorded from a specifically designed questionnaire or staff survey.

To assess PA and sedentary behavior, the activity tracker Fitbit Zip (Fitbit Inc: San Francisco, CA, USA) was used. The Fitbit Zip has a small, convenient size, and can be attached to clothing with a clip. The long battery lifetime of up to 6 months makes it suitable for use in long-term studies. Recently among others, Fitbit devices have been validated for use in community-dwelling older adults [45,46,47], rehabilitation, and cancer patients [48,49]. Step counts are the primary measure of activity trackers. In this context, it has been shown that Fitbit delivers data that are by far better than subjective measures and close to that of the “gold standard” of ActiGraph [50].

Participants were continuously recruited into the project depending on access to the nursing homes. Nursing home residents were asked to wear the Fitbit Zip for at least 10 days at their hip or necklace during waking hours. Participants and nursing staff were instructed to apply the Fitbit Zip immediately after getting up and to take it off before going to bed. Each device was programmed with the respective age, height, and weight of the participant. The nursing staff were briefed to remind participants to wear the activity tracker daily. For synchronization of the activity data, participants were visited every week by a study nurse. Participants were able to read the display of the Fitbit, but were not provided targeted feedback of their daily PA behavior.

*Physical activity behavior* was analyzed as steps per day (daily steps). Therefore, hours with at least one step were identified as wearing hours [39]. Data of days with either less than six hours of wearing the Fitbit Zip (identified as six hours with at least one step) or not fulfilling the “three a day criterion” (using the tracker at least once in the morning, once around noon, and once in the afternoon, identified as at least one step has been registered) were excluded and not used for the calculation of PA behavior. These days were classified as non-wearing days, according to the heuristic of Meyer and colleagues [51].

*Sedentary minutes* were determined as periods of at least 20 min with three or less steps per minute [50]. The “*longest zero*” describes the longest uninterrupted period of the day at which zero steps were taken. To calculate the sedentary time and longest zero, data of days with less than six wearing hours were excluded.

To express the sedentary minutes as a percentage of the total waking time, *waking time* was defined as a period of 12 h [52]. This arbitrary definition of waking time was necessary, as non-wearing and sedentary time are difficult to distinguish [51], that is, hours with zero steps either occur due to not wearing the tracker at all or due to sedentary time. There was only one participant who had a sedentary time of, on average, more than 12 h per day (Mean = 824.7 sedentary minutes/day), but was scaled down to 12 h.

Participants wore the tracker between 12 and 185 days, as they joined the project at different times. Further intermediate breaks or premediate interruptions influenced total wearing days. Due to these different wearing days of the individual participants, PA and sedentary behavior was evaluated for three different time periods: (1) for the entire period (over the entire wearing time of each participant), (2) during summer (over at least 2 weeks in July and/or August as the majority of the participants wore the tracker during this time) and (3) for the first seven wearing days (during the first seven days after the individual starting period of the participants). For pre-post analysis, a period of five weeks was used, as the majority of the subjects (*n* = 19) wore the Fitbit Zip for at least five weeks.

*Usage behavior* was identified on the basis of wearing days and classified as the frequency of days worn the tracker in relation to the participation period. Usability, acceptability and motivational impact of the activity tracker were assessed by use of an adapted version of the German-language version of the MeCue questionnaire [53]. We used six items—two items were adapted to assess the participants’ perception of the tracker’s ability to motivate them to be more physically active (“Activity tracker motivates me to do more physical activity”; “Activity Tracker with Feedback would motivate me to do more physical activity”), two items to assess the ease of use (“Activity tracker is easy to use (e.g., attaching on clothes)”; “I could have easily handled the device by myself (e.g., requesting steps)”), and one item to assess acceptance of the tracker (“Activity tracker is annoying”). The first five item-responses were recorded on a 5-point Likert scale (with 1 = strongly disagree to 5 = strongly agree). The last item recorded the overall experience with the Fitbit Zip by a visual analogue scale (VAS, 0–10).

### 2.3. Statistical Analysis

Activity tracker data were extracted by a dedicated system developed within the PROMOTE study of the AEQUIPA project [54,55]. The system extracted the activity tracker data from the Fitbit databases with the JSON-based Fitbit public API. Minute-wise data were available through the intra-day API. The resulting data were then analyzed locally in our own system. We investigated steps as the primary measure of the activity tracker for assessing PA and sedentary behavior. 

Descriptive information was generated for PA data (daily steps) and sedentary behavior (sedentary minutes, longest zero), for usage behavior of the activity tracker and for usability, acceptability, and motivation (answers of the questionnaire) of the participating nursing home residents in IBM SPSS Statistics (Version 26.0. IBM Corp. Armonk, NY, USA). Descriptive data are presented as frequencies, percentage, means, and standard deviations. As the data of the PA (daily steps) and sedentary behavior (sedentary minutes, longest zero) were not normally distributed, non-parametric tests were used for analysis. Statistical differences between age (<85 vs. ≥85 years), gender, usage behavior (<50% and ≥50% wearing time), cognition (no vs. mild or moderate dementia), current participation in nursing home activities (never or rare vs. frequently or daily), and past activity level (never or rare vs. occasionally or regularly) were evaluated by using the Mann-Whitney U-Test. For pre-post analyses, differences of steps, sedentary minutes, longest zero, and usage behavior between the first and fifth wearing week were assessed with the paired sample Wilcoxon test. The Wilcoxon test was also used to compare wearing periods (entire period, summer, first seven days). 

## 3. Results

As mentioned above, data of 22 long-term nursing home residents were analyzed. The main subject’s characteristics are presented in Table 1.

### 3.1. Daily PA of Nursing Home Residents

Participants wore the activity tracker for, on average, 76.59 ± 54.67 days (range 12 to 185 days).

#### 3.1.1. Daily Steps

**Figure 1:**
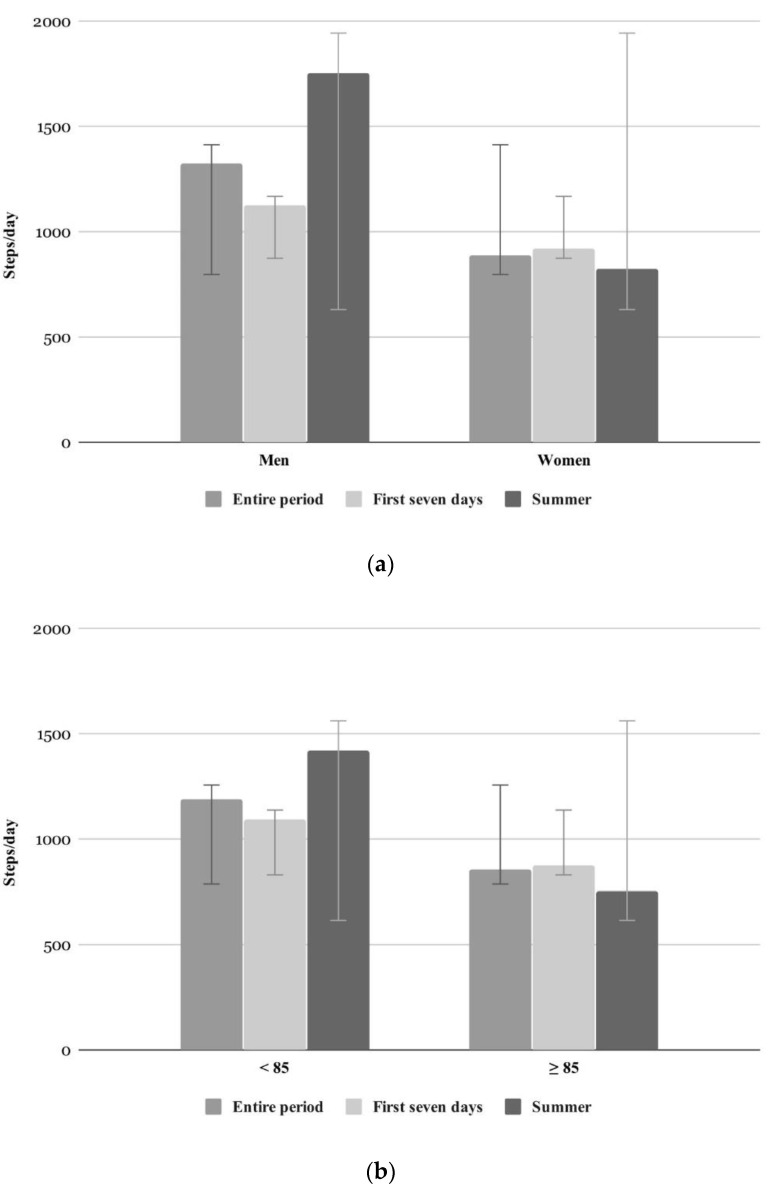
Daily steps of different time periods (entire period (*n* = 22), first seven days (*n* = 22), summer months (*n* = 20)) separated by (**a**) gender and (**b**) age.

**Figure 2:**
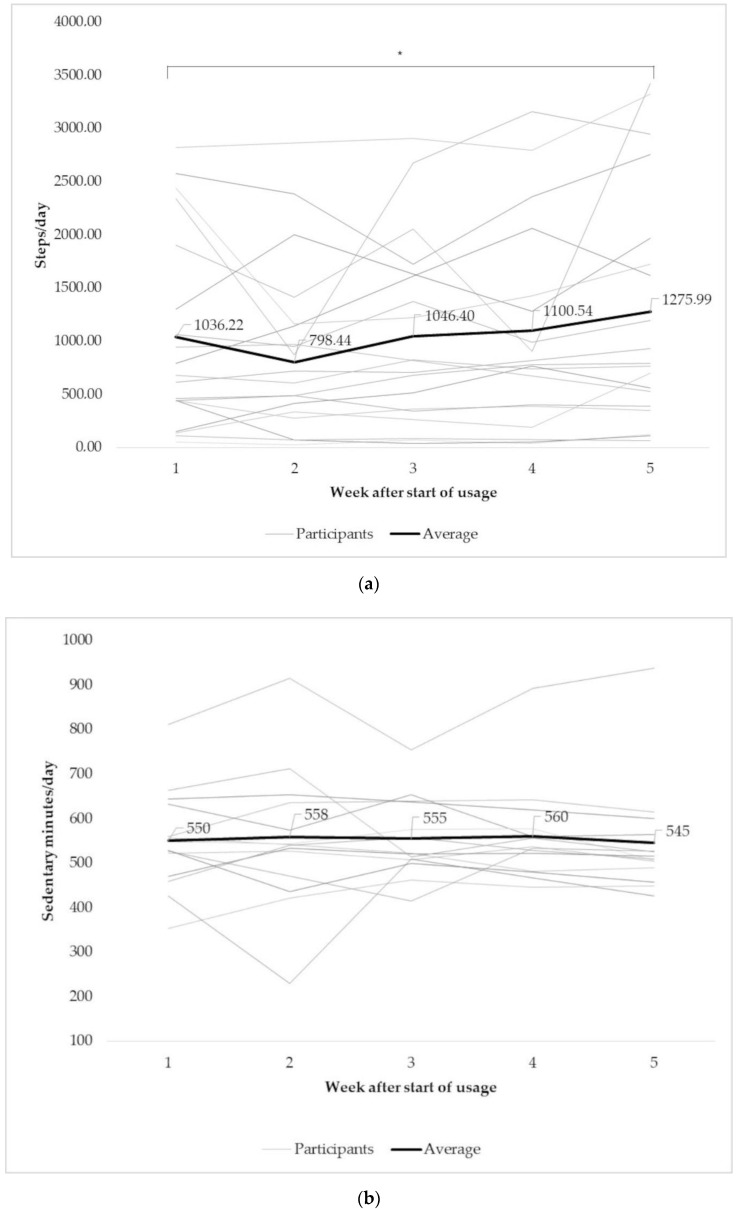

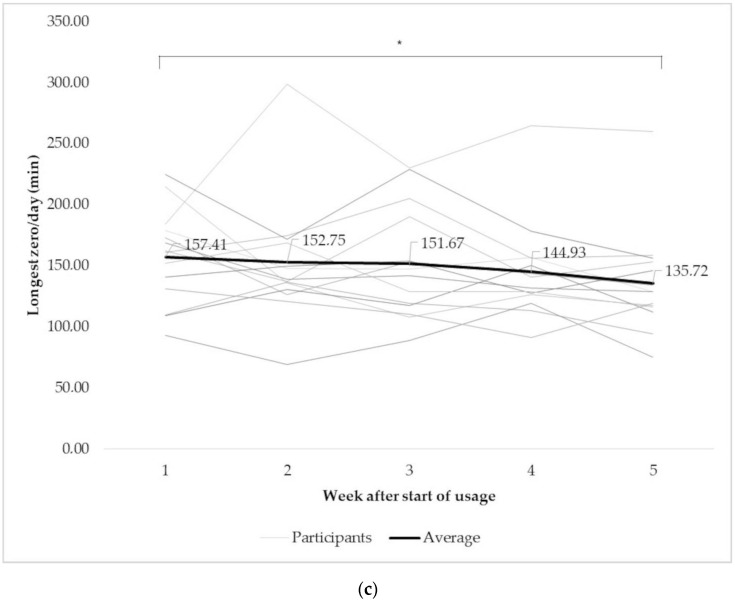
Average of (**a**) weekly number of steps/day, (**b**) weekly sedentary minutes/day, and (**c**) weekly longest zero/day over the period of the first five weeks of wearing the Fitbit Zip (*n* = 19) for the whole group (black line) and each single participant. Significant differences between the first and fifth week are marked by * (cf. (**a**,**c**)).

**Figure 3:**
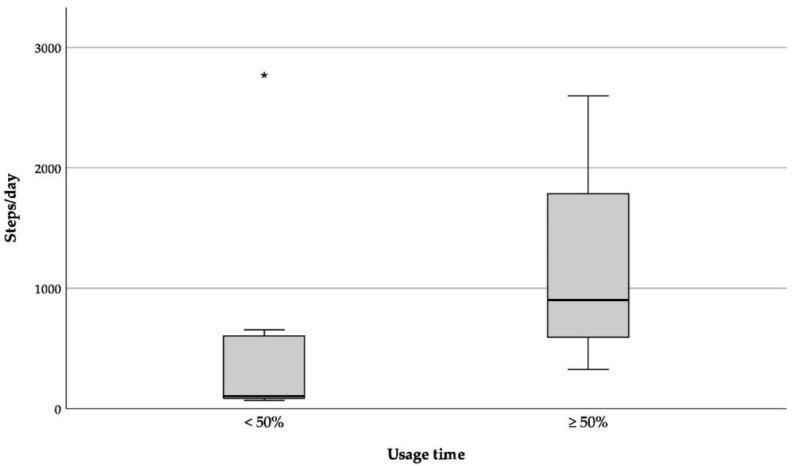
Average of daily steps separated by relative usage time of the Fitbit Zip (*n* = 22). * = outlier.

According to the Fitbit Zip data, the participants walked an average of 1007 ± 860 steps per day over their individual wearing period (range 70 to 2770 steps per day). During the first seven days of wearing the Fitbit Zip, participants walked, on average, 974 ± 877 steps per day (range 53 to 2821 steps per day). During summer, participants (*n* = 20) walked, on average, 1055 ± 933 steps per day (range 61 to 2930 steps per day; cf. Table 2). As shown by the paired sample Wilcoxon test, there were no significant differences in daily steps between the three analyzed periods (entire period, first seven days, and summer). Although, descriptively, men performed more steps then women and younger more than older participants, the steps taken per day did not differ significantly between gender and age groups (cf. Figure 1, Table 4). This was true for all analyzed periods. The results, however, showed that significantly more steps were taken in the fifth week of wearing than in the first (cf. Table 3 and Figure 2a). Participants who wore the activity tracker for at least 50% of their wearing period showed a significantly higher number of steps per day than participants who wore it for less than 50% of the study period (cf. Table 3 and Figure 3).

Further steps per day during the entire period did not differ significantly depending on cognition status (no (M = 998.7 ± 948.5 steps per day) vs. mild or moderate dementia (M = 1075.6 ± 898.1 steps per day)) and past activity level (never or rare (M = 849.0 ± 711.4 steps per day) vs. occasionally or regularly (M = 1138.0 ± 978.5 steps per day)). Currently active participants (frequently or daily active (M = 1269.9 ± 862.3 steps per day)), however, revealed significantly more steps per day than non-active ones (never or rarely active (M = 304.5 ± 254.4 steps per day); z = −2.6; *p* = 0.01).

#### 3.1.2. Daily Sedentary Behavior

To analyze the daily sedentary behavior, the data of participants with, on average, less than 6 h of wearing time were excluded. The average of the longest period of daily sedentary time without interruption (with zero steps, i.e., longest zero) amounted to 154.6 ± 37.4 min with a range from 94.3 to 247.6 min (*n* = 17; women: 160.6 ± 40.1; men: 140.1 ± 28.3, cf. Table 2). 

During the entire study period, participants (*n* = 17) spent, on average, 561.7 ± 86.3 min (9.36 h) per day sedentary (range 444.6–824.7 min (7.4–13.7 h), cf. Table 2). In relation to an assumed wearing time of 12 h, the participants spent, on average, 77.16 ± 9.47% of their time sedentary (range 62%–100%) and, on average, 21.47 ± 5.20% of their day uninterrupted sitting or lying down (range 13.0–34.0%). The results showed no significant differences in sedentary minutes between the first and fifth wearing week (*n* = 14; cf. Table 3, Figure 2b). However, the longest zero was significantly lower in the fifth than the first week of wearing (*n* = 14; cf. Table 3, Figure 2c). During the first seven days of wearing time, participants (*n* = 16) spent, on average, 541.0 ± 113.9 min sitting or lying down (352.2–810.9 min) and 152.3 ± 37.7 min sedentary without interruption (*n* = 16; 79.2–225.0 min, Table 2). During summer, participants (*n* = 17) spent an average of 568.1 ± 42.5 min per day sedentary (501.2–632.2 min) and showed a longest zero of 156.4 ± 31.8 min (116.4–274.2 min, Table 2). Sedentary minutes did not differ significantly between the three analyzed periods, but participants showed a significantly higher longest zero during summer compared to the entire period (z = −2.2; *p* = 0.02).

**Figure 4:**
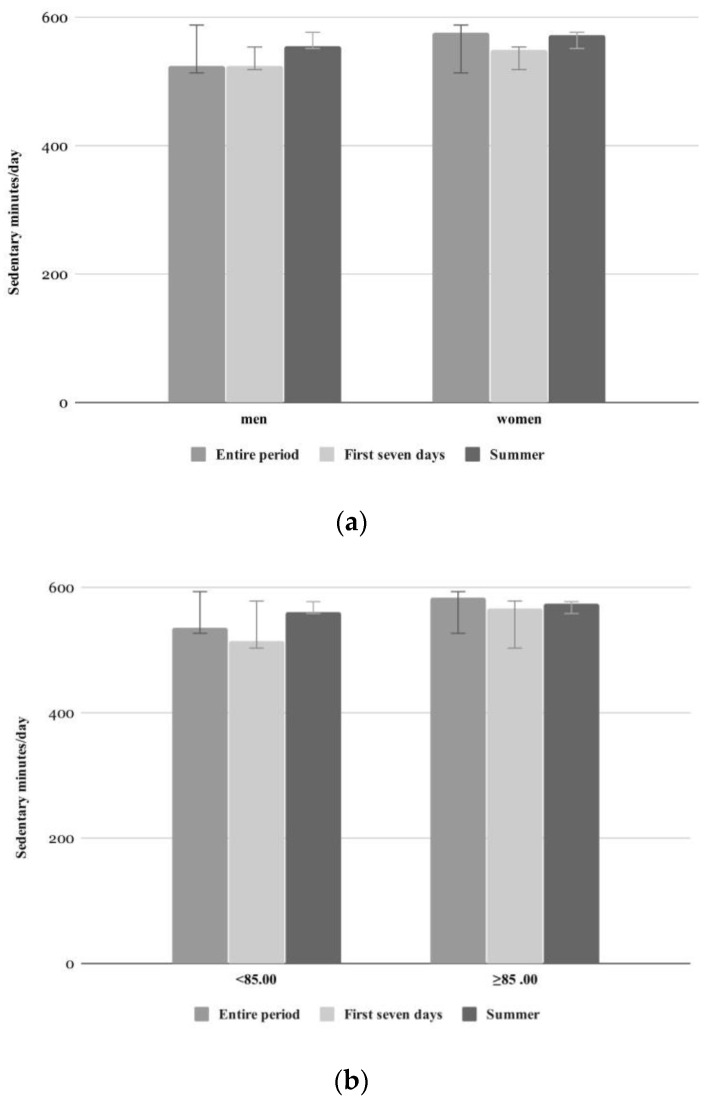

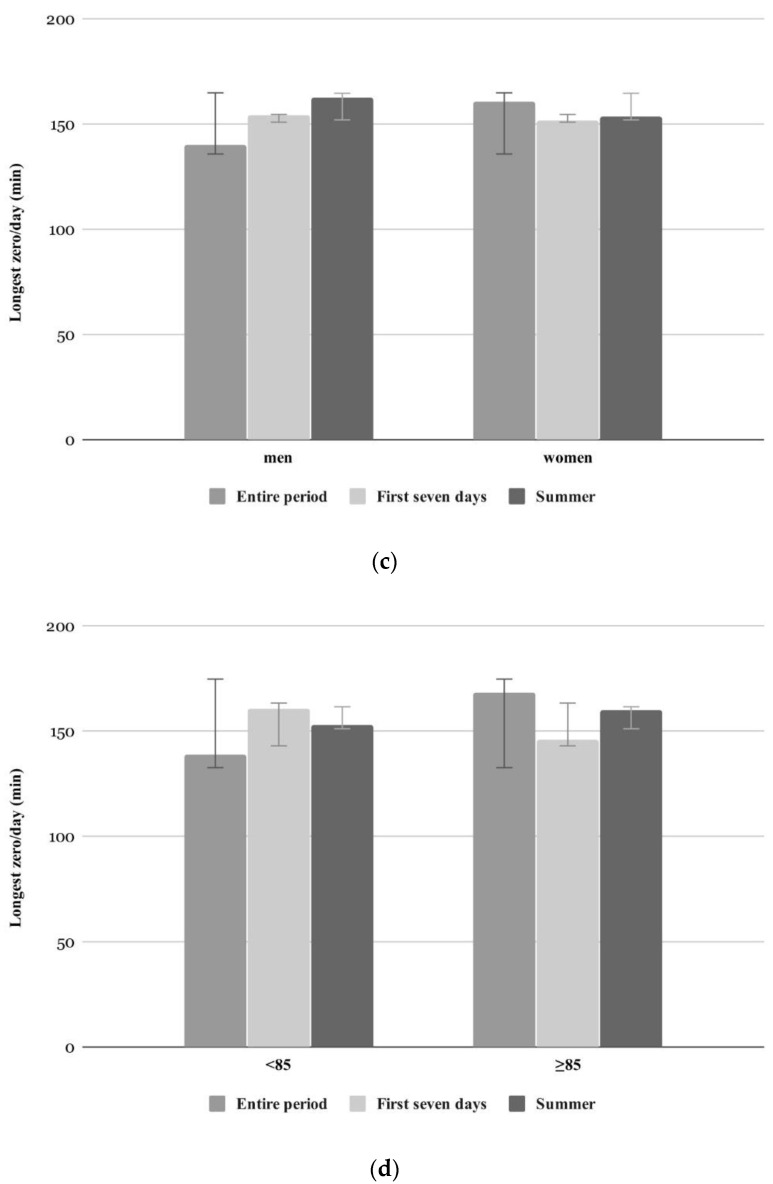
Daily sedentary behavior shown as sedentary minutes (**a**,**b**) and longest period of minutes with zero steps (longest zero, (**c**,**d**)) of different time periods (entire period (*n* = 17), summer (*n* = 17), first seven days (*n* = 16)) separated by gender (**a**,**c**) and age (**b**,**d**).

There were no significant differences of sedentary behavior (sedentary minutes, longest zero) for the entire period between men and women or different age groups (cf. Table 4 and Figure 4). The same was true with respect to the cognition status (no (M = 586.5 ± 139.3 min) vs. mild or moderate dementia (M = 548.7 ± 43.5 min)) or current (never or rarely (M = 595.5 ± 24.1 min) vs. frequently or daily active (M = 554.5 ± 93.6 min)) and past activity level (never or rare (M = 596.3 ± 108.6 min) vs. occasionally or regularly (M = 537.5 ± 61.2 min)). This also applied for the first seven days of wearing the Fitbit Zip and during summer.

### 3.2. Usability, Acceptability and Motivational Capacity of Activity Trackers for Nursing Home Residents

**Figure 5:**
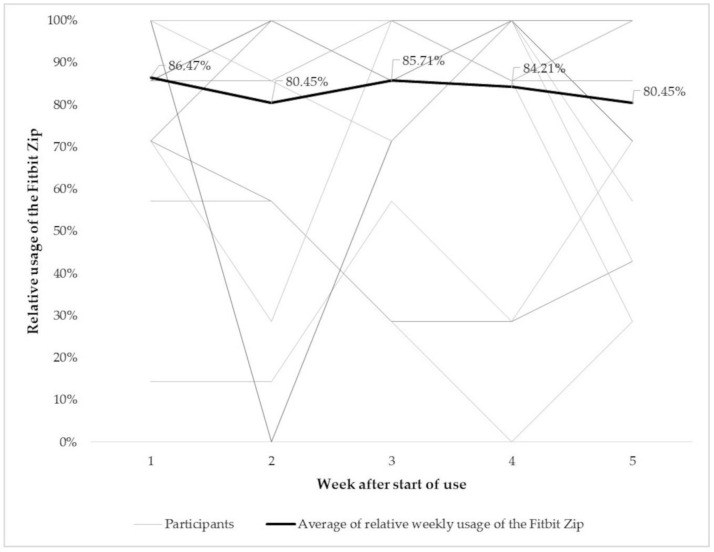
Average of the relative weekly usage of the Fitbit Zip over the period of the first five weeks of wearing (*n* = 19).

**Figure 6:**
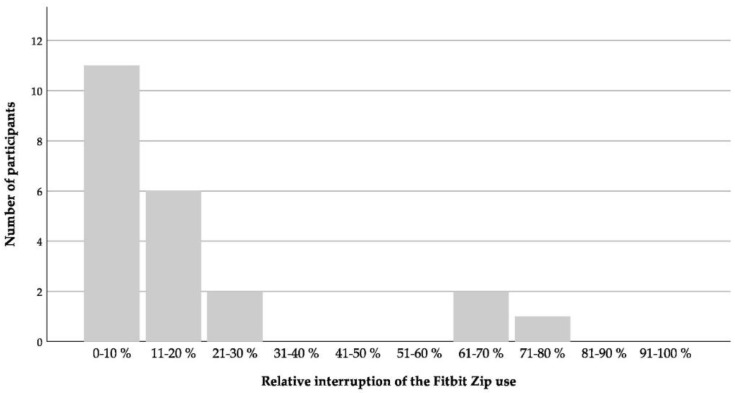
Relative interruption of the Fitbit Zip use during the individual wearing period (*n* = 22).

Further, usage behavior (*n* = 22), usability, acceptability, and motivational impact (*n* = 18) of the activity trackers were evaluated. From 31 recruited participants, five (16.7%) wore the Fitbit Zip for less than 10 days. The 22 included participants wore the activity tracker at least 6 h or three times a day for, on average, 65.4% of their individual study period and for, on average, 83.4% for the first five wearing weeks (Figure 5). There were no significant differences in usage time between the first and fifth wearing week (Table 3 and Figure 5), despite a higher amount of steps in week five (cf. Table 3). Twenty participants (90.9%), who wore the Fitbit Zip longer than 10 days, temporarily or prematurely interrupted their wearing time. The temporary interruption took, on average, 17 ± 19 days (3–64 days) and 20.5 ± 22.9% (3–79%) of the individual study period (Figure 6). Individual unpredictable reasons for different interruptions are shown in Table 5.

From the 22 participants that were included into the analysis of the Fitbit data, four (18.2%) were not able or not willing to answer the questionnaire. The results of the questionnaire are presented in Table 6 and Table 7. Of the participants, 94.4% reported that they accepted the activity tracker, and 33.3% of the participants reported that the Fitbit Zip was not easy to use, while 50% of the participants indicated good usability of the Fitbit Zip (e.g., attaching of the activity tracker, handling). Of the participants, 38.9% did not agree to the statement that they were able to handle the Fitbit Zip without problems, while 44.4% agreed (e.g., request information about steps). Of the participants, 77.8% indicated that just wearing the activity tracker (without feedback) did not change their daily activity behavior, and 38.9% reported that activity trackers with feedback would motivate them to do more PA. The overall experience with the activity tracker (0–10 points) was positive and rather good, at 6.95 points (range 5–10).

## 4. Discussion

This pilot and feasibility study assessed, first, the PA level of nursing home residents and, second, the feasibility of activity trackers for this vulnerable population across a wearing period up to 185 days. The results of the present study suggest that nursing home residents have a very low activity level of about 1000 steps per day with a range between 70 and 2770 steps, and spend about 9 h (with a minimum of nearly 7.5 and a maximum of 12 h) of their waking time sedentary, usually without interruptions. Furthermore, we were able to show that it is feasible and well-accepted to apply activity trackers in the nursing home setting and that usage behavior remains high, even over a longer wearing period. The results also indicate that wearing activity trackers in itself can motivate the wearer to take more steps.

Our data revealed that elderly nursing home residents had a very low level of daily activity. Participants walked, on average, 1007 ± 860 steps per day during their individual study period (12–185 days), which is nearly consistent with, but even lower than, the findings from Buckinx and colleagues (1678.4 ± 1621 steps/day), who examined the ambulatory PA level of nursing home residents over a period of seven days [20]. The results are far below the commonly recommended 10,000 steps per day [56], although these general recommendations are possibly non-transferable to a multi-morbid, frail population of nursing home residents. All participants of our study were multi-morbid, but able to walk independently with or without a walking aid and able to understand and execute simple instructions. In this context, Tudor-Locke and colleagues translated public health recommendations in terms of steps/day to special populations and older adults, and suggested an average of 1200–8000 steps/day for special populations (cancer, coronary heart diseases, diabetes, COPD, joint or muscle disorders) and 2000–9000 steps/day for healthy older adults [57]. As a mean, our participants did not reach the lowest threshold, and even the most active participants (2770 steps/day) only slightly came within the recommendations for older adults. There is, however, no sufficient evidence that allows developing guidelines and recommendations for daily steps in older adults, and especially not for elderly dependents like nursing home residents. 

Interestingly, the average number of steps/day increased significantly from the first to the fifth week of wearing. In addition, the nursing home residents who wore the Fitbit Zip for at least 50% of their individual study period took significantly more steps/wearing time than those with a shorter wearing time. The higher number of steps came along with a significant reduction of uninterrupted sedentary behavior (longest zero) but no significant difference in total sedentary minutes (only 1 min difference). These results indicate that besides being more active, participants might have improved their amount of steps per minute, that is, improved their physical performance.

The results suggest that using activity trackers can positively influence the PA behavior of nursing home residents, even without targeted feedback. In this context, several studies have shown that interventions aiming to promote walking (e.g., through activity trackers) can increase PA behavior in sedentary and older community-dwelling individuals [7,32,33,58].

Descriptively, men and younger older adults showed higher activity levels. However, these differences were not significant. This might be due to the very high variability within the sample. Men’s tendency to be more active proved significant in other studies [59,60,61,62,63] that showed that men spent more time in moderate to vigorous PA. These studies, however, examined the PA of community-dwelling older adults, which differs from the institutional setting analyzed in this study. “Traditional gender roles” and individual lifestyles might lead to differences in daily PA by gender in community-dwelling adults. Probably, men may be involved in more vigorous and outdoor activities, like gardening, while women are more likely to engage in lighter activities, such as housework [64]. Men and women living in nursing homes might reveal more similar daily routines, such as the same eating times, daily care, and group activities. The higher activity level in the younger of the old participants might be an indicator of age-related decline in physical performance and, in turn, PA level. Age differences in activity level and sedentary behavior were, however, not significant. These results are in contrast to other studies which have examined sedentary behavior in free living conditions and showed that sedentary behavior increased with older age [60,62,64], and that women were less sedentary and spent more time in moderate PA than men [59,63]. Even this difference could be traced back to the widespread practice of traditional gender roles in community-dwelling older adults.

There were no significant differences in sedentary minutes between the three analyzed time periods (whole period, summer, first seven days). However, the higher amount of the longest zero in summer compared to the entire period may indicate a seasonal effect. As the data of the entire period include the data of summer, no direct conclusions can be drawn. For analyzing seasonal or weather effects in future studies, data of different weather and seasons should be compared. Further, activity trackers with integrated GPS could be used to draw conclusions about the outside movement.

Our nursing home residents spent, on average, more than two and a half hours sitting or lying down without interruptions (21.5% of their waking time) and more than nine hours, or 77.2%, of their waking time sedentary. These findings are similar to or even higher than results of other studies, which examined sedentary behavior under free living conditions in older adults (74.5%, 65%, and 66% of wear time) [59,63,64], and also similar to or a bit lower than studies which examined sedentary behavior of nursing home residents (79% and 85% of wear time) [18,65]. The small discrepancies might be due to differences in (1) facility-organized activities, (2) regional differences of the examined nursing homes, (3) physical limitations of the residents, or (4) the devices that the activity was measured with. Nevertheless, these results are alarming, knowing the negative consequences of a sedentary lifestyle. Past research has shown that sedentary behavior is related to negative health outcomes, loss of physical function, and even increased mortality [66,67,68]. Reducing sedentary minutes could, in turn, promote one’s health status and physical functioning [69,70]. In this group of older adults living in nursing homes, approaches for prevention in long-term care are getting increasingly important. Simonsick and colleagues showed that even a small amount of regular walking can protect from further mobility loss [71]. To avoid negative health outcomes of already multi-morbid, frail older nursing home residents, sedentary time, especially uninterrupted sedentary periods, should be reduced and daily PA should be increased. Offers integrated into daily life, such as increasing daily walking, might be a feasible option, especially for residents who are not able to or not interested in participating in group activities. In order to develop common guidelines for the PA of vulnerable older adults, further research is needed.

The second objective of the study was to examine usage behavior, usability, acceptability, and the motivational impact of activity trackers. The usability of the Fitbit Zip can be considered good, with 65.4% usage time. With more than 83% usage time, the willingness to wear the Fitbit Zip was especially high during the first five wearing weeks. Despite this high level of commitment, the activity trackers were not used consistently. Over 90% of the participants temporarily or entirely suspended usage. However, temporary usage gaps represented, on average, 20.5% of the individual study period. In addition to the aforementioned reasons (forgot to apply, hospitalization, lost interest, temporarily lost the activity tracker), the design of the Fitbit Zip may have had an influence on usage (e.g., color, type, size) [72]. In previous research, Reid and colleagues investigated the feasibility of using the activePAL3^TM^ for aged residential care residents for a time period of seven days, and showed that 83.9% of the participants wore their trackers without interruptions. In relation to the current results, studies of younger populations showed higher dropout or abandonment rates during long-term examination of wearable activity trackers [51,73,74]. It has, however, to be mentioned that 16.7% of the 31 recruited participants were excluded from the analysis because of a wearing time of less than 10 days, and nearly 10% because of other factors (lost the Fitbit Zip and the consequent data loss; deceased). Nevertheless, about 71% (22 of 31 participants) of the recruited sample used the tracker over a longer period of time. Given the observed high commitment, long-term use of activity trackers for nursing home residents can be assumed as feasible. Nonetheless, actionable steps have to be taken to keep the level of commitment high in the long run.

Based on the questionnaire, nearly 95% of the participants accepted the Fitbit Zip. Related studies reported a positive acceptance of similar devices in community-dwelling older and/or impaired adults [35,37,42,75]. According to the questionnaire results, the Fitbit Zip has moderate usability for nursing home residents (mean 3.39/5 and 3.06/5). Half of the participants attested to good usability (e.g., attaching on clothes), while nearly 34% reported that the tracker was not easy to use. This result is in contrast to Rosenberg and colleagues who showed that 93% of the participating men with prostate cancer (mean age = 70.5 years) found the Fitbit Zip easy to wear and comfortable [75]. These discrepancies may result from differences in the study populations. Since women represent the majority in the present sample, difficulties with attaching the Fitbit Zip to skirts or dresses should be noted. Sensory, visual, or cognitive impairments of nursing home residents could also hamper easy use of the Fitbit Zip. Arm lace-shaped activity trackers might be easier to use for older adults living in long-term care institutions. The results of the usability questionnaire also suggest that technical operations should be performed by others (e.g., staff members). Another option would be the adaption of tracking devices to the limitations of nursing home residents.

About 80% of the study participants reported that solely wearing the Fitbit Zip did not change their daily activity behavior. However, these findings are inconsistent with the measured PA patterns given in steps per day that significantly increased between week one and five. Nearly 40% agreed with the statement that targeted feedback would motivate them to be more physically active. Studies that examined the impact of targeted feedback (e.g., via PA dairy or display on the activity tracker) on daily steps have shown a motivational effect of activity trackers for community-dwelling older adults and/or vulnerable populations [7,31,32,33,42]. Previous research has further shown that usage of activity trackers can improve functional status and health conditions of elderly people [7,25,76]. It has to be mentioned that the results of the current study cannot be fully compared with others, particularly because of the questionnaire which was specifically adapted for nursing home residents. Further reasons for the lack of comparability might be differences in (1) age groups, (2) activity tracker devices, (3) study period, or (4) location. All these factors might have a substantial impact on acceptability, usability, and motivational impact of the activity trackers. The feasibility of activity tracker usage for (dependent) older adults should be given more attention in further studies.

One limitation of our pilot study was the small sample size, which restricts generalization and subgroup analyses. In addition, we only estimated a daily wearing time of 12 h, and the validity of the determined ratio of sedentary minutes per day is restricted. An accurate recording of daily wearing time of the activity devices should be considered in future studies. The inclusion criteria of the nursing home residents constitute another limitation, especially the criterion to be able to walk independently. The nursing home residents with the most severe frailty were likely excluded, and therewith, the results of daily PA may have even been overestimated. Furthermore, several studies have proven that the validity of activity trackers is generally lower at low gait speed [21,77,78]. Nevertheless, devices of the Fitbit brand have been validated for use in community-dwelling older adults [45,46,47], rehabilitation, and cancer patients [48,49]. Given the increasing use of activity trackers to assess PA and sedentary behavior in all age and social groups, there is a high need for further validations.

## 5. Conclusions

The major strength of our study was the implementation of activity trackers for a longer period of time of up to 185 days to assess PA of a vulnerable population, as well as the assessment of the feasibility of such devices in a long-term care setting. So far, few studies have investigated these parameters for such a population, and if so, the variables were analyzed separately and/or only for a short period of time [18,19,20]. In addition, to ensure a high level of compliance, the nursing staff was integrated into the study process and supported the residents during the investigation period (e.g., attaching the tracker, assistance in case of loss). The results of the present study are alarming, looking at the very low activity levels and high sedentary time of nursing home residents. However, based on the results of the usage behavior and the questionnaire, it can be assumed that the application of activity trackers in care institutions is well-suited to analyze and increase PA behavior over a longer period of time. In particular, the institutional framework of nursing homes might contribute to good feasibility. Due to the moderate usability results, more customized devices like wristbands should be considered. Furthermore, the collaboration with facility management and especially nursing staff is essential. Involvement of staff members in the study design process could enhance compliance [19], especially for cognitively impaired participants. With regard to the low PA level and high feasibility of activity trackers for the participating nursing home residents, such devices should be used more widely in the field of nursing homes or older age groups, respectively. Further investigations are needed to develop general recommendations and interventions for this highly vulnerable population.

## Figures and Tables

**Table 1:** Characteristics of the population (*n* = 22; Mean ± SD OR number (%)).

Characteristic	Mean ± SD OR Number (%)				
	Whole Group (*n* = 22)	<85 (*n* = 10)	≥85 (*n* = 12)	Men (*n* = 6)	Women (*n* = 16)
Age (years)	86.4 ± 9.3	78.4 ± 6.6	93.1 ± 4.9	79.3 ± 8.5	89.1 ± 8.4
Gender (women)	16 (72.7)	5	11	6	16
Cognitive status					
No dementia (MoCA >23 OR staff survey = “no dementia”)	8 (36.4)	3	5	2	6
Mild dementia (MoCA = 18–23 OR staff survey = “mild dementia”	8 (36.4)	4	4	3	5
Moderate dementia (MoCA 10–17 OR staff survey = “moderate dementia”)	4 (18.2)	2	2	1	3
Missing	2 (9.1)	1	1	-	2
Height (cm)	162.3 ± 9.6	165.2 ± 9.5	159.7 ± 9.4	170.2 ± 12.2	159.2 ± 6.4
Weight (kg)	69.0 ± 17.4	75.7 ± 14.7	63.5 ± 18.1	76.3 ± 15.8	66.3 ± 17.7
BMI (kg/m²)	26.4 ± 6.0	27.7 ± 4.7	25.2 ± 7.0	26.4 ± 5.0	26.4 ± 6.5
Current level of activity					
never	3 (13.6)	2	1	1	2
rarely	3 (13.6)	0	3	1	2
frequently	7 (31.8)	2	5	1	6
daily	9 (40.9)	6	3	3	6
Past level of activity					
never	4 (18.2)	1	3	1	3
rarely	6 (27.3)	4	2	2	4
occasionally	5 (22.7)	2	3	1	4
regularly	7 (31.8)	3	4	2	5

**Table 2:** Daily physical activity (PA) of the entire period, first seven days, and during summer (two weeks in July and/or August).

	*n*	Mean (SD)	Min	Max
Steps entire period	22	1007.0 (860)	70.0	2770.0
Steps summer	20	1055.0 (933)	61.0	2930.0
Steps first seven days	22	974.0 (877)	53.0	2821.0
Sedentary minutes entire period	17	561.7 (86.3)	444.6	824.7
Sedentary minutes summer	17	568.1 (42.5)	501.2	632.2
Sedentary minutes first seven days	16	541.0 (113.9)	352.2	810.9
Longest zero entire period	17	154.6 (37.4)	94.3	247.6
Longest zero summer	17	156.4 (31.8)	116.4	274.2
Longest zero first seven days	16	152.3 (37.7)	79.2	225.0

**Table 3:** Steps per day and wearing time (usage time (%)) separated by wearing week (first vs. fifth wearing week) and usage behavior (<50% vs. ≥50% usage).

Wearing Week	First Wearing Week Mean (SD)	Fifth Wearing Week Mean (SD)	Wilcoxon-Test *z*-Value; *p*-Value
Steps (*n* = 19)	1020 (973)	1276 (1115)	−2,1; 0.04 *
Sedentary minutes (*n* = 14)	550.9 (122.5)	549.9 (113.5)	−0.31; 0.98
Longest zero (*n* = 14)	157.4 (38.4)	135.7 (42.7)	−2.1; 0.03 *
Usage time (%; *n* = 19)	85.1 (24.4)	80.5 (27.0)	−0.9; 0.37
**Relative Usage Time of the Fitbit Zip**	**<50%**	**≥50%**	**Mann-Whitney U-Test** ***z*-Value; *p*-Value**
Steps	617.7 (980.4)	1188.1 (766.4)	−2.1; 0.04 *

* *p* < 0.05.

**Table 4:** Difference between age groups and gender for daily PA level.

Age	<85 Years Mean (SD)	≥85 Years Mean (SD)	Mann-Whitney U-Test *z*-Value; *p*-Value
Steps entire period	1188.0 (905)	856.0 (829)	−0.7; 0.54
Steps summer	1423.0 (1069)	754.0 (720)	−1.1; 0.30
Steps first seven days	1093.0 (870)	875.0 (908)	−0.8; 0.46
Sedentary minutes entire period	536.8 (51.7)	583.8 (106.7)	−1.0; 0.34
Sedentary minutes summer	561.4 (40.5)	574.8 (46.5)	−0.7; 0.48
Sedentary minutes first seven days	514.5 (93.2)	567.5 (132.0)	−0.6; 0.53
Longest zero entire period	138.8 (23.6)	168.6 (42.9)	−1.6; 0.29
Longest zero summer	152.7 (23.7)	160.1 (40.0)	−0.3; 0.75
Longest zero first seven days	160.4 (29.3)	146.0 (43.8)	−0.7; 0.49
**Gender**	**Men** **Mean (SD)**	**Women** **Mean (SD)**	**Mann-Whitney U-Test** ***z*-Value; *p*-Value**
Steps entire period	1323.0 (1009)	888.0 (801)	−1.0; 0.33
Steps summer	1752.0 (1140)	823.0 (761)	−1.8; 0.08
Steps first seven days	1126.0 (833)	918.0 (912)	−0.7; 0.54
Sedentary minutes entire period	524.5 (56.7)	577.2 (93.6)	−1.2; 0.25
Sedentary minutes summer	555.3 (39.6)	573.2 (44.5)	−0.7; 0.48
Sedentary minutes first seven days	523.9 (125.3)	548.8 (113.8)	−0.1; 1.00
Longest zero entire period	140.1 (28.3)	160.6 (40.1)	−0.4; 0.67
Longest zero summer	162.8 (23.3)	153.8 (35.4)	−1.3; 0.20
Longest zero first seven days	154.1 (34.8)	151.5 (40.5)	−0.1; 0.96

**Table 5:** Reasons for temporarily interruptions of the individual wearing period.

Reason for Temporary Interruption or Premature Abortion *n* = 20	Number (%)
Forgot to apply	11 (55)
Hospitalization	1 (5)
Lost Interest	4 (20)
Lost Fitbit Zip (temporarily)	4 (20)

**Table 6:** Acceptability, usability, motivational capability of Fitbit Zip—Mean Scores.

Item	Mean (SD)				
	Whole Group (*n* = 18)	<85 (*n* = 9)	≥85 (*n* = 9)	Men (*n* = 5)	Women (*n* = 13)
Acceptability (1–5 *)					
Activity tracker is annoying	1.27 (0.75)	1.11 (0.33)	1.44 (1.01)	1.00 (0.00)	1.38 (0.87)
Usability (1–5 *)					
Activity tracker is easy to use (e.g., attaching on clothes)	3.39 (1.20)	3.67 (1.22)	3.11 (1.17)	3.40 (1.14)	3.38 (1.26)
Personal handling of the activity tracker without problems	3.06 (0.94)	3.00 (1.00)	3.11 (0.93)	3.00 (1.00)	3.08 (0.95)
Current motivation (1–5 *)					
Activity tracker motivates me to do more physical activity	1.78 (0.81)	1.67 (0.87)	1.89 (0.78)	2.00 (1.00)	1.69 (0.75)
Potential Motivation (1–5 *)					
Activity tracker with feedback would motivate me to do more physical activity	3.45 (1.15)	3.00 (1.00)	3.89 (1.17)	3.40 (1.14)	3.46 (1.20)
Overall experience (0–10 °)					
Overall experience of the activity tracker	6.95 (1.55)	7.00 (1.58)	6.89 (1.62)	6.80 (1.10)	7.00 (1.73)

* 1 = strongly disagree, 2 = disagree, 3 = neither agree nor disagree, 4 = agree, 5 = strongly agree; ° using a visual analogue scale (0 = bad; 10 = good).

**Table 7:** Acceptability, usability, motivational capability of Fitbit Zip—Frequencies.

Item	Strongly Disagree/Disagree N (%)	Neither Agree Nor Disagree N (%)	Strongly Agree/Agree N (%)
Acceptability			
Activity tracker is annoying	17 (94.4)	0 (0)	1 (5.6)
Usability			
Activity tracker is easy to use (e.g., attaching on clothes)	6 (33.3)	3 (16.7)	9 (50.0)
Personal handling of the activity tracker without problems	7 (38.9)	3 (16.7)	8 (44.4)
Current motivation			
Activity tracker motivates me to do more physical activity	14 (77.8)	4 (22.2)	0 (0.0)
Potential Motivation			
Activity tracker with feedback would motivate me to do more physical activity	4 (22.2)	7 (38.9)	7 (38.9)
